# Subject level clustering using a negative binomial model for small transcriptomic studies

**DOI:** 10.1186/s12859-018-2556-9

**Published:** 2018-12-12

**Authors:** Qian Li, Janelle R. Noel-MacDonnell, Devin C. Koestler, Ellen L. Goode, Brooke L. Fridley

**Affiliations:** 1 Department of Biostatistics and Bioinformatics, Moffitt Cancer Center, 12902 Magnolia Drive, Tampa, FL 33612 USA; 2 Health Informatics Institute, University of South Florida, Tampa, FL USA; 3 Children’s Mercy Hospital, Kansas City, MO USA; 4 Department of Biostatistics, University of Kansas Medical Center, Kansas City, KS USA; 5 Department of Health Sciences Research, Mayo Clinic, Rochester, MN USA

**Keywords:** Negative binomial, Model-based, RNA-seq, EM algorithm, Clustering, Gaussian mixture model

## Abstract

**Background:**

Unsupervised clustering represents one of the most widely applied methods in analysis of high-throughput ‘omics data. A variety of unsupervised model-based or parametric clustering methods and non-parametric clustering methods have been proposed for RNA-seq count data, most of which perform well for large samples, e.g. *N* ≥ 500. A common issue when analyzing limited samples of RNA-seq count data is that the data follows an over-dispersed distribution, and thus a Negative Binomial likelihood model is often used. Thus, we have developed a Negative Binomial model-based (NBMB) clustering approach for application to RNA-seq studies.

**Results:**

We have developed a Negative Binomial Model-Based (NBMB) method to cluster samples using a stochastic version of the expectation-maximization algorithm. A simulation study involving various scenarios was completed to compare the performance of NBMB to Gaussian model-based or Gaussian mixture modeling (GMM). NBMB was also applied for the clustering of two RNA-seq studies; type 2 diabetes study (*N* = 96) and TCGA study of ovarian cancer (*N* = 295). Simulation results showed that NBMB outperforms GMM applied with different transformations in majority of scenarios with limited sample size. Additionally, we found that NBMB outperformed GMM for small clusters distance regardless of sample size. Increasing total number of genes with fixed proportion of differentially expressed genes does not change the outperformance of NBMB, but improves the overall performance of GMM. Analysis of type 2 diabetes and ovarian cancer tumor data with NBMB found good agreement with the reported disease subtypes and the gene expression patterns. This method is available in an R package on CRAN named *NB.MClust*.

**Conclusion:**

Use of Negative Binomial model based clustering is advisable when clustering over dispersed RNA-seq count data.

**Electronic supplementary material:**

The online version of this article (10.1186/s12859-018-2556-9) contains supplementary material, which is available to authorized users.

## Background

A common goal of RNA-seq studies is unsupervised clustering [1–3]. Unsupervised clustering analysis has been widely used to group samples to determine ‘latent’ molecular subtypes of disease or to cluster genes into modules of co-expressed genes, where within which each of the clusters observations are more similar to one another than those in other clusters. The goal of this study is to develop a unsupervised cluesting method for that takes into account the over-dispersed nature of RNA-seq count data for the clustering of samples. Popular unsupervised clustering methods include non-parametric methods, such as, K Means, Nonnegative Matrix Factorization (NMF), hierarchical clustering [4] and parametric methods, such as, Gaussian mixture modeling (GMM) or Gaussian model-based (MB) clustering), which model the data as coming from a distribution that is mixture of two or more components [5–9]. In the context of model-based clustering there are challenges in applying standard model-based clustering to RNA-seq data, including the discrete nature of the data and the over-dispersion observed in the data (i.e., the variance is greater than the mean).

Over the past decade a substantial body of research has emphasized the importance of over-dispersion in the statistical modeling of RNA-seq data. Durán Pacheco et al. [10] compared the performance of four model-based methods for treatment effect comparison on over-dispersed count data in simulated trials with different sizes of clusters (i.e. number of individuals in each cluster) and correlation level within each cluster. They found important performance impact on group comparison testing aroused by cluster sample size and accounting for over-dispersion. The over-dispersion characteristic in discrete count data can be captured by Negative Binomial (NB) or Poisson-Gamma (PG) mixture density. A flexible NB generalized linear model for over-dispersed count data was proposed by Shirazi et al. [11] with randomly distributed mixed effects characterized by either Lindley distribution or Dirichlet Process (DP). Si et al. [12] derive a Poisson and Negative Binomial model-based clustering algorithms for RNA-seq count data to group genes with similar expression level per treatment, using Expectation-Maximization (EM) algorithm along with initialization technique and stochastic annealing algorithm. Other methods such as nonparametric clustering are also widely employed in latest research [6, 13–15], but cannot guarantee a consistent and accurate result for certain cases. For example, NMF clustering results may vary tremendously due to the randomness of starting point [16], and hierachical clustering performance depends on distance metric or linkage [17].

Gaussian Model-Based clustering with logarithm or Blom [18] transformation often works well for some discrete data. However, these transformations still show limitations in capturing the over-dispersed nature of RNA-seq data [19, 20]. Therefore, we developed a model-based clustering approach that accounts for the over-dispersion in RNA-seq counts by using a mixture of Negative Binomial distributions, denoted by NBMB. The clustering methods proposed by Si et al. [12] are model-based for either Poisson or Negative Binomial data, but restricted to grouping of genes based on limited number of samples from different treatments.

Similar to the algorithms by Si et al. [12], we combined the EM algorithm with stochastic annealing to fit the Negative Binomial model-based clustering algorithm. In developing the algorithm, we propose a more efficient technique for estimating dispersion parameter and initialization of parameters. Another concern with this application of the EM algorithm is the use of annealing rates. Several stochastic algorithms have been proposed and applied in existing research involving EM algorithm, see [21, 22]. Research by Si et al. [12] applied both deterministic and simulated annealing algorithms and follow the suggested rate values by Rose et al. [21]. However, these values might not be able to provide global optimal prediction in some circumstances for our model. Simulated scenarios in this research illustrate that slight adjustments in annealing rate can avoid local or less optimal prediction and bring improvement on performance of the Negative Binomial model-based clustering algorithm. Hence, in our algorithm we search and locate the optimal annealing rates for NBMB on RNA-seq raw counts. In the following sections, we describe the details of the proposed NBMB method, along with assessment of the method with an extensive simulation study based on an RNA-seq data from the TCGA study of ovarian cancer. Lastly, we present results from the application of NBMB and GMM to two studies; an obesity and type 2 diabetes study and an ovarian cancer study conducted by TCGA.

## Methods

### NBMB clustering method

The observed RNA-seq data set *X* is a *N* × *G* matrix, where *N* is the sample size and *G* is the number of genes being considered for the clustering analysis. Each row of *X* represents RNA-seq gene counts for a sample, denoted by *x*_*i*_, *i* = 1, …, *N*, where each element of *x*_*i*_ is denoted by *x*_*ig*_, *i* = 1, …, *G*. In this study we assume independence between all genes and independence between all samples or subjects. Suppose the samples of RNA-seq counts *x*_1_, …, *x*_*N*_ can be grouped into *K* clusters. NBMB assumes *x*_*i*_ belongs to cluster *k*, *k* = 1, …, *K*, and *x*_*i*_~*NB*(*μ*_*k*_, *θ*_*k*_), where *μ*_*k*_ = (*μ*_*k*1_, …, *μ*_*kG*_), *θ*_*k*_ = (*θ*_*k*1_, …, *θ*_*kG*_) are the parameters of cluster *k*. The density function of *x*_*ig*_ is$f\left(\left.{x}_{ig}\right|{\mu}_{kg},{\theta}_{kg}\right)=\frac{\varGamma \left({x}_{ig}+{\theta}_{kg}\right)}{\varGamma \left({\theta}_{kg}\right){x}_{ig}!}{\left(\frac{\mu_{kg}}{\theta_{kg}+{\mu}_{kg}}\right)}^{x_{ig}}{\left(\frac{\theta_{kg}}{\theta_{kg}+{\mu}_{kg}}\right)}^{\theta_{kg}}$. The value of *k* for each sample *x*_*i*_ is unknown and cannot be observed. The prior probabilities for each sample belonging to each component are *p*_1_, …, *p*_*K*_ and *p*_1_ + … + *p*_*K*_ = 1. The log likelihood function for *x*_1_, …, *x*_*N*_ is $L=\sum \limits_{i=1}^N\log \left({\sum}_{k=1}^K{p}_kf\left(\left.{x}_i\right|{\mu}_k,{\uptheta}_k\right)\right)$, where *f*(*x*_*i*_|*μ*_*k*_, θ_*k*_) is the density function for sample *i* when it is assigned to cluster component *k*; *p*_*k*_ is the prior probability for belonging to component *k*; *μ*_*k*_, θ_*k*_ are the mean and dispersion of component *k*. The NBMB method assumes that RNA-Seq counts without batch and the library size effects follow the NB distribution, hence, NBMB must be applied to normalized counts without any transformation.

### Estimation

We adopt the following EM algorithm to optimize likelihood function *L* and estimate *μ*_*k*_ and classification of samples. The optimal value of K is determined by Bayesian Information Criterion (BIC) [7, 23].

#### E-step

Calculate subject-specific posterior probability and value of expectation function.

The expectation function at iteration (*s* − 1) is given by:

$$ {l}_k^{\left(s-1\right)}\left({\mu}_k^{\left(s-1\right)}\right)={\sum}_{i=1}^N{p}_{ik}^{\left(s-1\right)}\log \left(f\left(\left.{x}_i\right|{\mu}_k^{\left(s-1\right)},{\widehat{\uptheta}}_k\right)\right), $$

where ${p}_{ik}^{\left(s-1\right)}$ is the posterior probability that sample *i* belongs to cluster component *k* at iteration (*s* − 1). In${p}_{ik}^{\left(s-1\right)}=\frac{p_k^{\left(s-1\right)}f\left(\left.{x}_i\right|{\mu}_k^{\left(s-1\right)},{\widehat{\uptheta}}_k\right)}{\sum_{k=1}^K{p}_k^{\left(s-1\right)}f\left(\left.{x}_i\right|{\mu}_k^{\left(s-1\right)},{\widehat{\uptheta}}_k\right)}$, ${p}_k^{\left(s-1\right)}$ is the prior probability for component *k* at iteration (*s* − 1);${\mu}_k^{\left(s-1\right)}$ is the mean of component *k* at iteration (*s* − 1) and $f\left(\left.{x}_i\right|{\mu}_k^{\left(s-1\right)},{\widehat{\uptheta}}_k\ \right)$ is the density function for sample *i* when it is assigned to component *k*.

#### M-step

Update *p*_*k*_ by ${p}_k^{(s)}=\frac{1}{N}{\sum}_{i=1}^N{p}_{ik}^{\left(s-1\right)}$and *μ*_*k*_ by maximizing${l}_k^{\left(s-1\right)}\left({\mu}_k^{\left(s-1\right)}\right)$, that is${\mu}_k^{(s)}=$ $\frac{\sum_{i=1}^N{p}_{ik}^{\left(s-1\right)}{x}_i}{\sum_{i=1}^N{p}_{ik}^{\left(s-1\right)}}$. The explicit form of${\mu}_k^{(s)}$ is derived by the first order gradient of${l}_k^{\left(s-1\right)}\left({\mu}_k\right)$. The estimate of dispersion ${\widehat{\uptheta}}_k$ is not updated throughout iterations, as explained in the following section.

### Modification of algorithm

#### Initialization

The model in Si et al. [12] use treatment information in initialization algorithm. NBMB adopt an initialization technique different from this algorithm as current study does not involve groups of genes. The initial value for the prior probability ${p}_k^{(0)}$ is set at *1/K*, while the initialization of mean and dispersion (${\mu}_k^{(0)},{\theta}_k^{(0)}$) are the MLE for all samples (denoted by${\widehat{\mu}}_{MLE}$,${\widehat{\theta}}_{MLE}$). A trivial shift in mean and dispersion between clusters can be adopted to avoid equivalent posterior probabilities at the first iteration, for example,$\kern0.50em {\mu}_k^{(0)}={\widehat{\mu}}_{MLE}\Big(1+0.01\left(k-1\right)$) and${\theta}_k^{(0)}={\widehat{\theta}}_{MLE}\left(1+0.01\left(k-1\right)\right)$, with shift step 0.01. This initial shift can be set to any value in R package *NB.MClust*, but large values may cause computation errors*.*

#### Dispersion estimate

The traditional M-step estimates *μ*_*k*_, *θ*_*k*_, *p*_*k*_ simultaneously by maximizing expected log likelihood function per component, without the closed form for estimate of *θ*_*k*_, which might lead to inefficient computation. A conclusion in Si et al. [12] states that treating *θ*_*k*_ as known via an estimated value in EM iterations does not affect the power of clustering. Therefore, we fix ${\widehat{\theta}}_k$ at initial values for all iterations. The estimate of dispersion in their work is the Quasi-Likelihood Estimate (QLE) proposed by Robinson et al. [24]. This technique significantly reduces computation time and avoid invalid values that can be produced at various iterations of the EM algorithm. We emloy the similar approach in the algorithm for NBMB using the exact MLE rather than the QLE over all samples, as Si et al. [12] found that a fixed value of the dispersion parameter in the EM algorithm did not impact the clustering results.

#### E-step rescaling and annealing

Due to the assumed independence between genes and subjects, we construct the multivariate density function *f*(*x*_*i*_|*μ*_*k*_, *θ*_*k*_) by multiplying G univariate Negative Binomial density functions$f\left(\left.{x}_i\right|{\mu}_k,{\theta}_k\right)={\prod}_{g=1}^G{f}_g\left(\left.{x}_{ig}\right|{\mu}_{kg},{\theta}_{kg}\right)$**.** However, the existence of zero value of *f*_*g*_(*x*_*ig*_|*μ*_*kg*_, *θ*_*kg*_) cannot be avoided when G is large (e.g. *G* ≥ 1000), which might result in the denominator of ${p}_{ik}^{\left(s-1\right)}$being zero. Setting the density function to an arbitrary nonzero value may lead to estimation errors for ${p}_{ik}^{\left(s-1\right)}$ and${p}_k^{(s)}$ . Thus, it is necessary to rescale the density function per sample per gene via dividing it by the exponential of mean density across all genes and clusters, that is changing *f*_*g*_(*x*_*ig*_|*μ*_*kg*_, *θ*_*kg*_) to${e}^{\log \left[{f}_g\left(\left.{x}_{ig}\right|{\mu}_{kg},{\theta}_{kg}\right)\right]-{M}_i}$, ${M}_i=\frac{1}{KG}{\sum}_{k=1}^K{\sum}_{g=1}^G\mathit{\log}\Big[{f}_g\left(\left.{x}_{ig}\right|{\mu}_{kg},{\theta}_{kg}\right).$ There have been several algorithms proposed to reduce the risk of local optimal solutions in EM algorithm, for instance, Simulated Annealing (SA) by [22] and Deterministic Annealing (DA) by [21]. We choose to use the DA algorithm to deal with issue of potential local optimum, hence, ${p}_{ik}^{\left(s-1\right)}$in the E-step is modified as${p}_{ik}^{\left(s-1\right)}=\frac{p_k{\left[f\left(\left.{x}_i\right|{\mu}_k^{\left(s-1\right)},{\theta}_k\right){e}^{-{M}_i}\right]}^{1/{\tau}_{s-1}}}{\sum_{k=1}^K{p}_k{\left[f\left(\left.{x}_i\right|{\mu}_k^{\left(s-1\right)},{\theta}_k\right){e}^{-{M}_i}\right]}^{1/{\tau}_{s-1}}}$ **.** In applying DA, we used the values *τ*_0_ = 2 *or* 10, *τ*_*s* + 1_ = *rτ*_*s*_ and *r* = 0.9, similar to the values proposed by Rose et al. [21], with *τ*_0_ = 10 providing an optimal solution in most of simulation scenarios. Assessment of the robustness via a simulation study found that these values do achieve the best performance across all scenarios.

### Simulation study

To assess the performance of NBMB, an extensive simulation study was completed in which NBMB was compared to GMM with no transformation, GMM on log transformed counts, and GMM on Blom transformed counts [25]. For each simulated data set, all the models were applied with number of cluster components K selected from K = 2, …, 6, based on BIC, with the clustering performance assessed with the Adjusted Rand Index (ARI) [26]. In this study we did not include K = 1 in the range of *K*, because we assumed at least two cluster components based on prior knowledge, similar to [8]. The simulation scenarios differ in terms of: number of clusters (K = 2, 3, 4, 5, 6); distance between clusters (*∆μ* = 0.1, 0.5, 1; *∆θ* = 0, 1 ); percentage of differentially-expressed (DE) genes (5, 10%); sample size (*N* = 50, 100, 150, 200); and number of genes (G = 1000, 5000). For each scenario, 100 datasets were generated. Model-based clustering usually requires filtering to a set of genes, as the performance is poor when too many ‘null’ genes (i.e., genes with trivial variation) are included. Recent gene expression studies often filtered genes down to 1000–5000 genes for clustering by some measure of variability [27, 28] or by a semi-supervised approach based on a clinical endpoint, such as overall survival [29, 30]. Hence, we considered the number of genes at *G*= 1000 and 5000.

The simulated data sets were generated by baseline parameters and the shift step between clusters. Baseline parameters values *μ*_1_ and *θ*_1_ were the MLE of *μ* and *θ* for the expression of the most variable genes in ovarian cancer tumor samples from TCGA. The first cluster component was generated by *μ*_1_ and *θ*_1_. The parameters for each of the remaining cluster components, i.e. *μ*_*k*_ and *θ*_*k*_, *k* = 2, …, *K* were computed as *μ*_*k*_ = *μ*_1_*e*^(*k* − 1)*∆μ*^, *θ*_*k*_ = *θ*_1_*e*^(*k* − 1)*∆θ*^, with *∆μ* and *∆θ* being the shift steps between two adjacent clusters. The shift step of mean *μ* was set to either a small, medium, or large effect (*∆μ* = 0.1, 0.5, 1), while the shift step of dispersion *θ* was set at either zero or one (*∆θ* = 0, 1). The value of *∆μ* was similar to the log fold change of RNA-Seq counts between the subgroups of TCGA ovarian cancer tumor samples.

### Application to real data

To assess the performance of NBMB to the commonly used GMM with transformations, the clustering methods were applied to two transcriptomic studies. For clustering analysis of each dataset, the most variable genes were selected based on the Median Absolute Deviation (MAD) as discussed in [31] and then used in the clustering analysis. We used the 1000 instead of 5000 genes with the largest variation based on MAD for the analysis of both data sets, as DE percentage for top 5000 genes is possibly smaller than that for top 1000 genes. The optimal number of clusters in each study was selected from a specified range of K based on the BIC criterion.

#### Obesity and type 2 diabetes study

The first application dataset is a longitudinal transcriptomic study of obesity and type 2 diabetes (T2D) in which RNA was extracted from isolated skeletal muscle precursor cells from 24 subjects. RNA was sequenced on the Illumina HiSeq 2000 platform, with data downloaded from Gene Expression Omnibus (GEO) at GSE81965**,** GSE63887. This dataset does not contain any known batch effects as described in [32]. We use *edgeR* package within R statistical software to calculate library size normalized counts based on the upper quartile normalization factor, followed by computation of the counts per million (CPM) for which clustering analysis was applied. The range of possible number of clusters (K) for the analysis were 2 to 5, since the four groups of subjects in the T2D study can be reclassified as case and control.

#### TCGA ovarian Cancer study

The second dataset contains normalized RNA-seq counts from the TCGA study of ovarian serous cystadenocarcinoma tumors (*N* = 295). Tissue site effect within this dataset is removed by Empirical Bayes method [33], with data downloaded from MD Anderson at http://bioinformatics.mdanderson.org/tcgambatch/ by selecting Disease ‘OV’, Center/Platform ‘illuminahiseq rnaseqv2 gene’, Data Level ‘Level 3’ and Data Set ‘Tumor-corrected-EBwithParametricPriors-TSS’. The downloaded data (*X)* was the normalized expression abundance on the log scale; therefore the data was converted to the original scale by *e*^*X*^. The range of possible clusters (K) was from 3 to 5, as multiple groups have reported that there are between 3 and 5 subtypes of serous ovarian cancer [34–36]. Cluster assignments from NBMB and GMM were compared to the CLOVAR subtypes, in which 4 subtypes have been described related to progression free survival [35].

## Results

### Simulation study

**Fig. 1:**
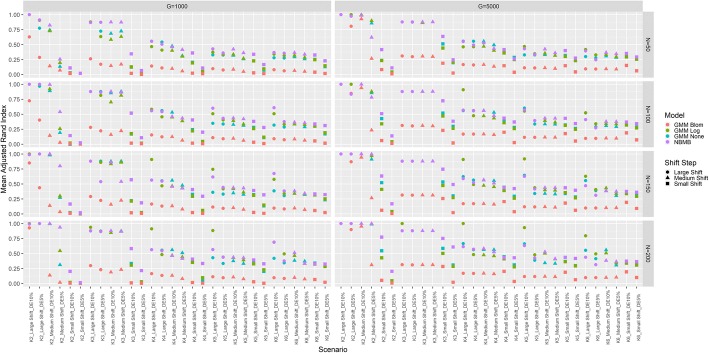
Simulation Results for Non-zero Shift in Dispersion. Plot of the mean Adjusted Rand Index for 100 simulated datasets in each of the scenarios with non-zero shift in dispersion parameter. Scenarios in each panel are ordered by K, shift step size, and DE percentage. Colors represent different methods, while shapes represent shift step size. Gaussian Mixture Model with none, log and Blom transformations are labeled as GMM None, GMM Log and GMM Blom

There were 480 simulation scenarios being assessed, with 100 datasets simulated per scenario. Unsupervised model-based clustering was completed on each simulated data set using the Negative Binomial mixture model (NBMB) or the Gaussian mixture model (GMM) with log, Blom or no transformation. To assess performance, adjusted rand index (ARI) was computed with mean ARI for the scenarios presented in Fig. 1 ( *∆θ* = 1), and Additional file 1: Figure S1 ( *∆θ* = 0). In general, NBMB outperformed the GMM in most of the scenarios, especially when total sample size or cluster distance is small. The simulation results also indicate that for the scenarios assessed, the none and logarithmic outperformed the Blom transformation for the Gaussian model-based approach.

Figure 1 illustrates that the performance metric ARI is decreasing as the number of clusters increases or the shift step size decreases. NBMB has better or equivalent performance compared to GMM on log transformed or raw data for limited sample sizes (*N* = 50, 100), regardless of the number of genes or the shift step in parameters, except for a few scenarios. This exception might be the result of simulation randomness or less optimal annealing rate. ARI of GMM on log transformed data increases sharply for the K > 3 scenarios at larger sample sizes (*N* = 150, 200) and higher proportion of DE genes (10%). The results in larger sample sizes also imply that GMM with log-transformation is an ideal approach for a large-scale study and may outperform NBMB in this scenario.

The contrasts between NBMB and GMM methods are consistent across the scenarios with different number of genes (G = 1000, 5000). Performance of these approaches also improved for certain scenarios when the number of genes changes from 1000 to 5000. However, this improvement does not imply that including more genes will definitely lead to better performance in the clustering of real data. If the percentage of DE genes decreases while still preserving the number of genes included in the clustering analysis, the clustering performance may decrease. Summary statistics for ARI per simulated dataset across different scenarios were listed in Additional file 2: Tables S1-S4.

### Analysis of obesity and type 2 diabetes study

**Fig. 2:**
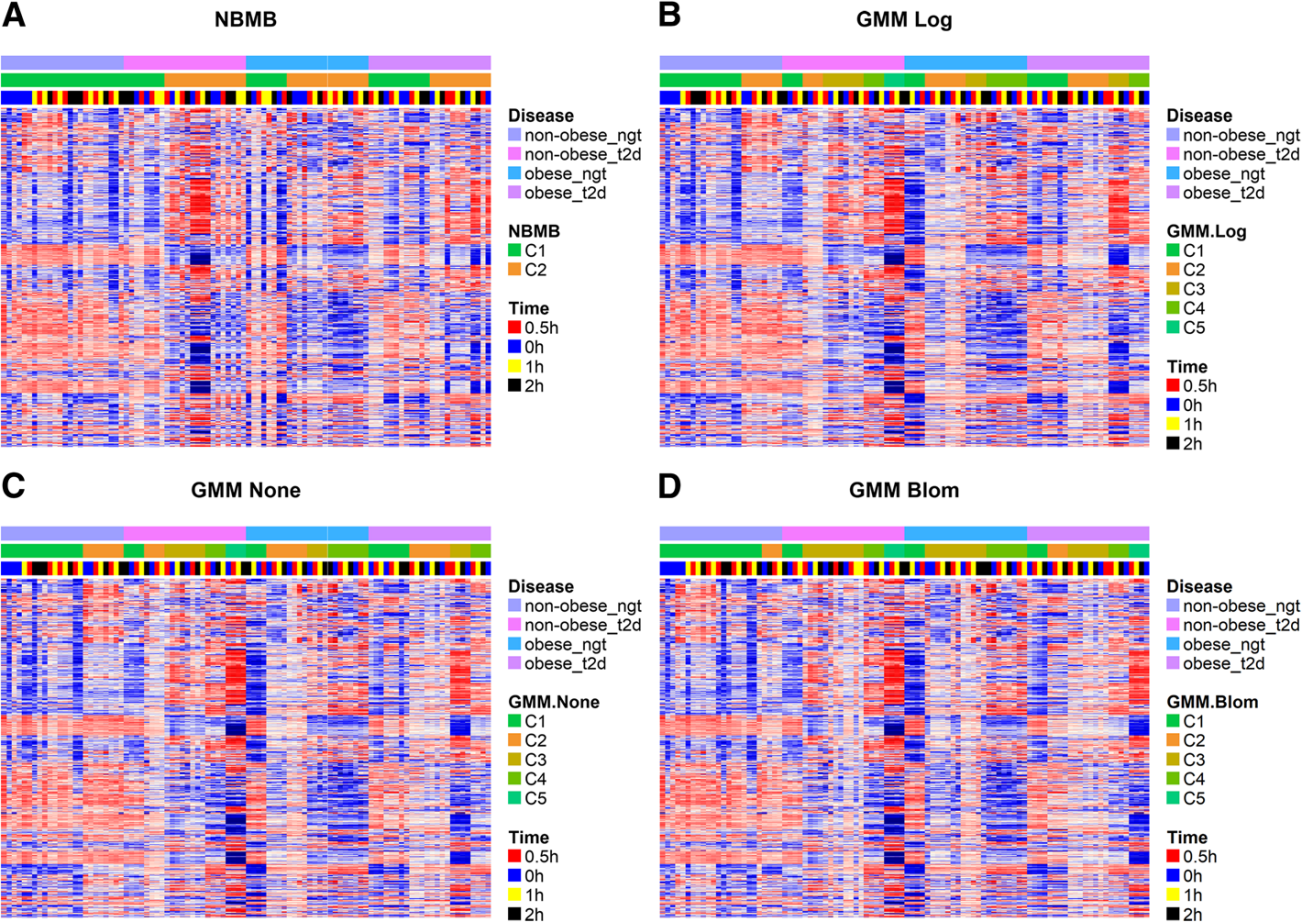
Heatmap for T2D Study: Ordered by Disease Subtypes. Heatmap of the 1000 top MAD genes for the 96 samples in Type 2 Diabetes study with disease subtypes and clustering results by (**a**) NBMB, (**b**) GMM log-transform, (**c**) GMM non-transformed, (**d**) GMM Blom-transformeddisplayed at the top. Rows represent genes and columns are subjects

In the Obesity and type 2 diabetes (T2D) study each subject was sampled at 4 time points: 0, 0.5, 1, and 2 h after insulin stimulation. Among the 24 subjects there are 6 normal glucose tolerant, 6 obese, 6 type 2 diabetic, and 6 obese and type 2 diabetic. We performed clustering by NBMB and Gaussian model-based methods on library size normalized RNA-seq counts of 96 samples and compared the clustering results to the known disease/phenotype groups. The comparison between the clustering methods is presented in Table 1 and Fig. 2. All the normal glucose tolerant subjects were clustered into NBMB cluster 1 (C1) and more than half of the T2D and/or obese subjects were assigned to NBMB cluster 2 (C2), matching with heatmap patterns in Fig. 2 (A). In contrast, the GMM with log, Blom and no transformation divide each disease/phenotype group into 2–5 subgroups with one cluster (C1 in each GMM method) overlapping with 2/3 or 5/6 of normal glucose tolerant samples (Table 1), but the remaining clusters are not overlapping with any other disease groups. The Fisher Exact test shows both NBMB and GMM clusters are significantly associated with disease groups.

**Table 1:** Overlap between Obesity and T2D disease groups and the unsupervised cluster assignments produced from GMM with or without transformation and NBMB

	Non-obese & Normal glucose tolerant	Non-obese & T2D	Obese & Normal glucose tolerant	Obese & T2D
GMM None				
C1	16 (66.7%)	4 (16.7%)	4 (16.7%)	8 (33.2%)
C2	8 (33.3%)	4 (16.7%)	8 (33.2%)	8 (33.2%)
C3	0	8 (33.2%)	4 (16.7%)	4 (16.7%)
C4	0	4 (16.7%)	8 (33.2%)	4 (16.7%)
C5	0	4 (16.7%)	0	0
GMM Blom				
C1	20 (83.3%)	4 (16.7%)	4 (16.7%)	4 (16.7%)
C2	4 (16.7%)	0	0	4 (16.7%)
C3	0	12 (50%)	12 (50%)	8 (33.4%)
C4	0	4 (16.7%)	8 (33.4%)	4 (16.7%)
C5	0	4 (16.7%)	0	4 (16.7%)
GMM Log				
C1	16 (66.7%)	4 (16.7%)	4 (16.7%)	8 (33.2%)
C2	8 (33.3%)	4 (16.7%)	8 (33.2%)	8 (33.2%)
C3	0	8 (33.2%)	4 (16.7%)	4 (16.7%)
C4	0	4 (16.7%)	8 (33.2%)	4 (16.7%)
C5	0	4 (16.7%)	0	0
NBMB				
C1	24 (100%)	8 (33.3%)	8 (33.3%)	12 (50%)
C2	0	16 (66.7%)	16 (66.7%)	12 (50%)

The heatmaps in Fig. 2 (a)-(d) also reveal the outperformance of NBMB according to the matching between the clusters and gene expression. The subjects in each heatmap were ordered first by disease subtypes and then by the clusters from each method. NBMB clusters in Fig. 2 (a) are consistent with the DE pattern in more than half of the selected genes. GMM on log and non-transformed data divide the non-obese normal glucose tolerant subjects into two subgroups (C1 and C2 in Fig. 2 (b)-(c)), only partially matching to the differential expression of a small number of genes. Similarly, the clusters given by GMM with Blom transformation do not show agreement with most of the patterns in Fig. 2 (d). Furthermore, the clustering result of NBMB implies that the difference between the non-obese subjects with normal glucose tolerance are less diverse compared to the other disease groups. NBMB cluster 2 (C2) identifies potential signatures for a subject having either obesity or T2D.

### Analysis of ovarian cancer study

**Fig. 3:**
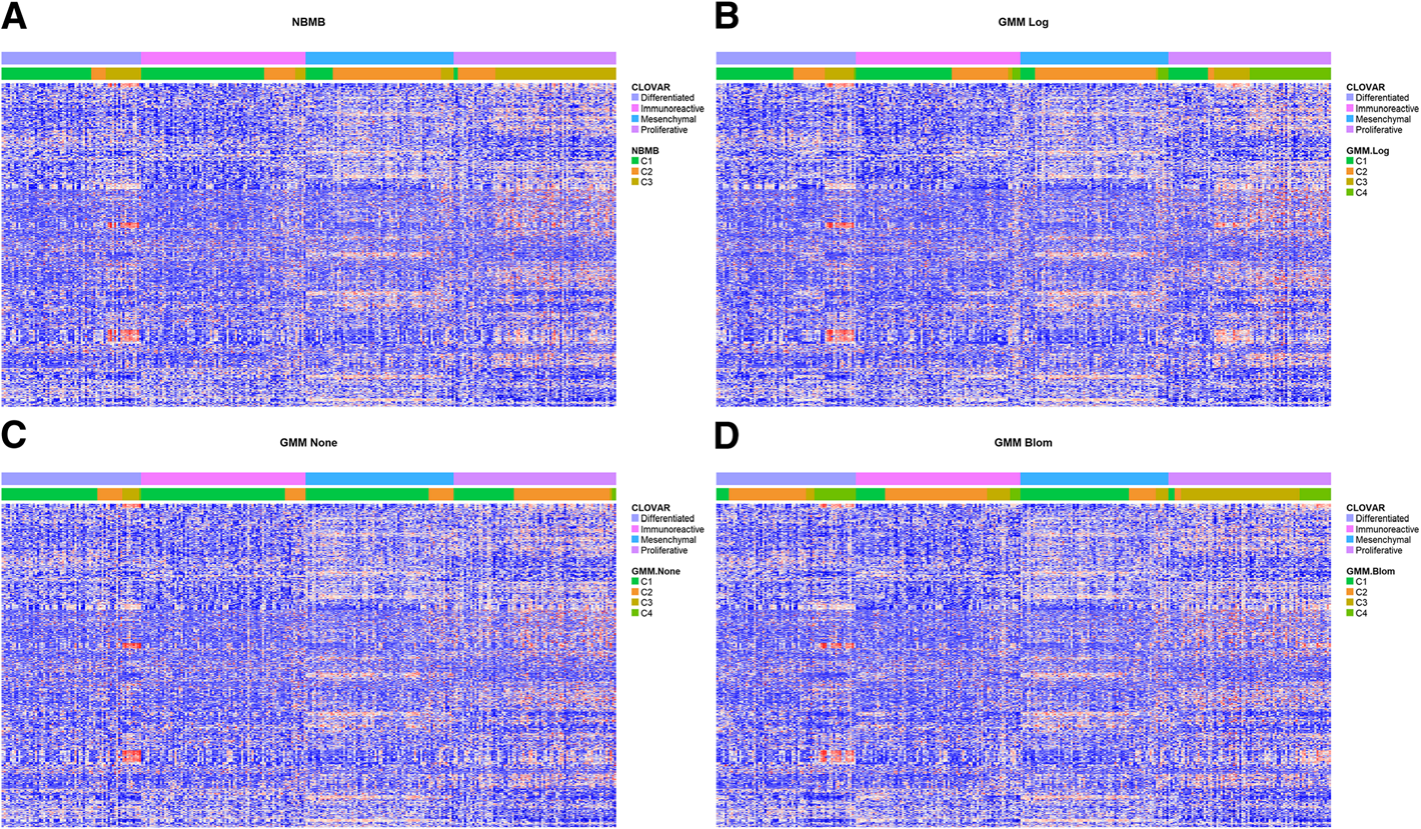
Heatmap for TCGA OV Study: Ordered by CLOVAR Subtypes. Heatmap of the 1000 top MAD genes for the 295 samples in TCGA ovarian cancer study with CLOVAR subtypes and clustering results by (**a**) NBMB, (**b**) GMM log-transform, (**c**) GMM non-transformed, (**d**) GMM Blom-transformed displayed at the top. Rows represent genes and columns are subjects

Research by TCGA [27] and Tothill et al. [37] found and defined four subtypes of high-grade serous ovarian cancer: Immunoreactive, Differentiated, Proliferative and Mesenchymal. These subtypes are later integrated with prognostic signatures and named as Classification of Ovarian Cancer (CLOVAR) framework by Verhaak et al. [38]. To assess NBMB and GMM with and without various transformations, we completed model-based clustering and compared these findings to the four CLOVAR subtypes, which are presented in Table 2 and Fig. 3. The CLOVAR subtypes used in this paper were determined by the single-sample Gene Sets Enrichment Analysis (ssGSEA) scores computed on the 100-gene CLOVAR signatures (see Additional file 2: Table S1 and S7 of [38]). NBMB determined 3 clusters, while GMM with different transformation found 4 clusters. NBMB cluster 1 (C1) was enriched for differentiated and immunoreactive subtypes, cluster 2 (C2) was enriched for mesenchymal and cluster 3 (C3) contained a large proportion of proliferative CLOVAR subtype samples. On the other hand, GMM completed on non-transformed, the Blom and log transformed count data show similar levels of agreement with the CLOVAR subtypes as the NBMB, with most of mesenchymal and proliferative samples clustered in different GMM clusters, i.e. C1, C3 of Blom transform and C2, C4 of log transform in Table 2. The Fisher Exact test shows that both NBMB and GMM clusters are significantly associated with CLOVAR subtypes.

**Table 2:** Overlap between CLOVAR subtypes and the unsupervised cluster assignments produced from GMM with or without transformation and NBMB

	Differentiated	Immunoreactive	Mesenchymal	Proliferative
GMM None				
C1	43 (68.25%)	85 (87.63%)	48 (87.27%)	27 (33.75%)
C2	11 (17.46%)	12 (12.37%)	7 (12.73%)	50 (62.5%)
C3	8 (12.7%)	0	0	1 (1.25%)
C4	1 (1.59%)	0	0	2 (2.5%)
GMM Blom				
C1	5 (7.94%)	22 (22.68%)	44 (80%)	4 (5%)
C2	38 (60.32%)	54 (55.57%)	8 (14.55%)	2 (2.5%)
C3	4 (6.35%)	12 (12.37%)	3 (5.45%)	59 (73.75%)
C4	16 (25.39%)	9 (9.28%)	0	15 (18.75%)
GMM Log				
C1	33 (52.38%)	52 (53.61%)	5 (9.1%)	19 (23.75%)
C2	16 (25.4%)	36 (37.11%)	47 (85.45%)	4 (5%)
C3	13 (20.63%)	4 (4.13%)	0	17 (21.25%)
C4	1 (1.59%)	5 (5.15%)	3 (5.45%)	40 (50%)
NBMB				
C1	37 (58.73%)	69 (71.13%)	10 (18.18%)	1 (1.25%)
C2	9 (14.29%)	21 (21.65%)	43 (78.18%)	19 (23.75%)
C3	17 (26.98%)	7 (7.22%)	2 (3.64%)	60 (75%)

The heatmaps in Fig. 3 (a)-(d) present the DE pattern for CLOVAR subtypes and the latent groups discovered by each method. Subjects in each heatmap were ordered first by CLOVAR and then by the clusters. NBMB and GMM log-transformed clusters in Fig. 3 (a)-(b) are consistent with the DE pattern in half of the selected genes, although GMM on log-transformed data divided NBMB cluster 3 (C3) into two subgroups, improving the agreement to heatmap DE pattern. This improvement confirms a conclusion in simulation study that GMM may outperform NBMB for large samples. Clusters identified by the other two GMM approaches severely deviated from the differential expression in Fig. 3 (c)-(d). In addition, NBMB cluster 1 (C1) in Fig. 3 (a) reveals the signatures accounting for similarity between differentiated and immunoreactive subtypes, while GMM log-transformed clusters 3 and 4 (C3, C4) in Fig. 3 (b) uncover a small number of DE genes for the latent groups in proliferative CLOVAR subtype.

It should be noted that the genes and methods used in the development of the CLOVAR subtypes are not exactly the same as those used in the application of NBMB and GMM methods. The CLOVAR subtypes are determined by the top 100 genes of the signatures in [27] that were consistently present in various ovarian cancer studies [35], while the NBMB and GMM methods perform unsupervised clustering with 1000 genes selected based on TCGA samples only. We do not use the CLOVAR signatures in NBMB and GMM methods, because our goal is to assess unsupervised clustering performance of model-based methods on high-dimensional RNA-Seq data rather than discovering unknown disease subgroups with the developed signatures.

## Discussion

In this paper, we presented a method and algorithm using a Negative Binomial mixture model (NBMB) to complete unsupervised model-based clustering of transcriptomic data from high-throughput sequencing technologies. In doing so, we assess the NBMB method using both an extensive simulation study and transcriptomic studies of type 2 diabetes and ovarian cancer. In general, we found that the NBMB outperforms Gaussian mixture model (GMM) applied to transformed data, particular when the same size was small or when difference in cluster means were small. In this study we choose to compare NBMB to other model-based approaches, as model-based methods are known to outperform nonparametric or heuristic-based methods when the correct model is specified [8, 39], especially when the range of number of clusters is known. Besides, Nonnegative Matrix Factorization (NMF) requires impractical computation time for large data matrices [15] and has limitation for certain distribution patterns [40], although it has been successfully used for transcriptome data analysis. Therefore, we only compare model-based methods for current research.

The strength of this research include the following: the ability to model the over-dispersion in transcriptomic data through the use of a Negative Binomial distribution for development of the mixture modeling framework for model-based clustering; the completion of an extensive simulation study to assess the performance of NBMB; the application of NBMB to two existing transcriptomic studies; and the ability for researchers to apply this method using the R package *NB.MClust*. The computation efficiency of NBMB, as implemented in R package *NB.MClust*, and GMM, as implemented in R package *mclust* with the function “Mclust”, was assessed by clustering the TCGA OV dataset with 295 samples and 1000 genes. User/system time (in seconds) for clustering with K = 3 for NB.MClust was 29.01/0.03, while Mclust had a time of 0.97/0.19. For the case when clustering was run for K selected from K = 3, 4, 5, the time (in seconds) for NB.MClust was 88.06/0.01, while Mclust had a time of 3.31/0.33. It should be noted that the package *mclust* was being well-developed and upgraded in computation for the past decades [7, 8, 41, 42].

In this study, the simulation results present a decrease in performance metric ARI for each method along with an increase in cluster components, as shown in Fig. 1. The reason for this trend is that adding more cluster components leads to smaller cluster sizes for a fixed total of samples, and consequently results in lower computation power. This performance change also sheds light on the range of K specified in model-based clustering methods for a given sample size. For example, for *N* = 50 the suggested range is K = 2, 3, 4 as the mean ARI by each method is below 0.5 for all K > 4 scenarios in Fig. 1. On the other hand, for a larger-scale study with *N* ≥ 150, it is necessary to include K = 5, 6 into the expected range of K in the application of NBMB or GMM with log transform. Hence, we used this guidance to set the range to select the optimal K in the clustering analysis of two transcriptomic studies. Furthermore, NBMB is superior to the other methods when a transcriptomic study contains no more than 3 subgroups. However, if there are K ≥ 4 subgroups in the T2D small-cohort study, the clusters given by each model-based method may deviate from the true subgroup assignment due to the limited sample size (*N* = 96). In contrast, if we expect K ≥ 4 subtypes in the TCGA OV large-scale study (*N* = 295), GMM on log-transformed data may be the optimal method to discover latent subtypes and the result is valid. The heatmap patterns in Fig. 2 (a) and Fig. 3 (b) illustrate that the T2D study contains two subgroups correctly identified only by NBMB, while the latent four subtypes of TCGA OV subjects are better discovered by GMM.

While this research provides a framework for model-based clustering of samples using a Negative Binomial distribution, future research is needed to extend NBMB in the following ways. First, research is needed to implement a gene selection approach within the NBMB model with a semi-surprised approach or a variable selection / shrinkage approach [30]. Secondly, research is needed into extending this model to the Bayesian framework wherein prior distributions could be used for the number of clusters or by modeling the number of clusters (i.e., infinite mixture model) as an infinite Dirichlet process [43]. Finally, further research is needed to extend the model to take into account the correlated nature of gene expression data.

## Conclusion

The NBMB clustering method fully captures the over-dispersion in RNA-seq expression and outperforms Gaussian model-based methods with the goal of clustering samples, particular when the sample size is small or the differences between the clusters (in terms of the mean) is small.

## Additional files

Additional file 1: **Figure S1.** Simulation Results for Zero Shift in Dispersion. Plot of the mean Adjusted Rand Index for 100 simulated datasets in each of the scenarios with zero shift in dispersion parameter. (PNG 246 kb)

Additional file 2: **Table S1.** Summary Statistics of ARI for G = 1000 and Non-zero Shift in Dispersion. **Table S2.** Summary Statistics of ARI for G = 5000 and Non-zero Shift in Dispersion. **Table S3.** Summary Statistics of ARI for G = 1000 and Zero Shift in Dispersion. **Table S4.** Summary Statistics of ARI for G = 5000 and Zero Shift in Dispersion (XLSX 129 kb)

## Abbreviations

ARI: Adjusted Rand Index
CLOVAR: Classification of Ovarian Cancer
CPM: Counts per million
DA: Deterministic Annealing
DE: Differentially expressed
EOC: Epithelial ovarian cancer
HGS: High grade serous
MAD: Median Absolute Deviation
MB: Model-Based
MLE: Maximum likelihood estimator
NBMB: Negative Binomial Model-Based
NMF: Nonnegative Matrix Factorization
QLE: Quasi-Likelihood Estimate
SA: Simulated Annealing
T2D: Type 2 Diabetes
TCGA: The Cancer Genome Atlas
