# Supplementary figures and images for: Subject level clustering using a negative binomial model for small transcriptomic studies
Source: BMC Bioinformatics. 2018 Dec 12;19:474. doi: 10.1186/s12859-018-2556-9 (PMC6292049; doi:10.1186/s12859-018-2556-9)

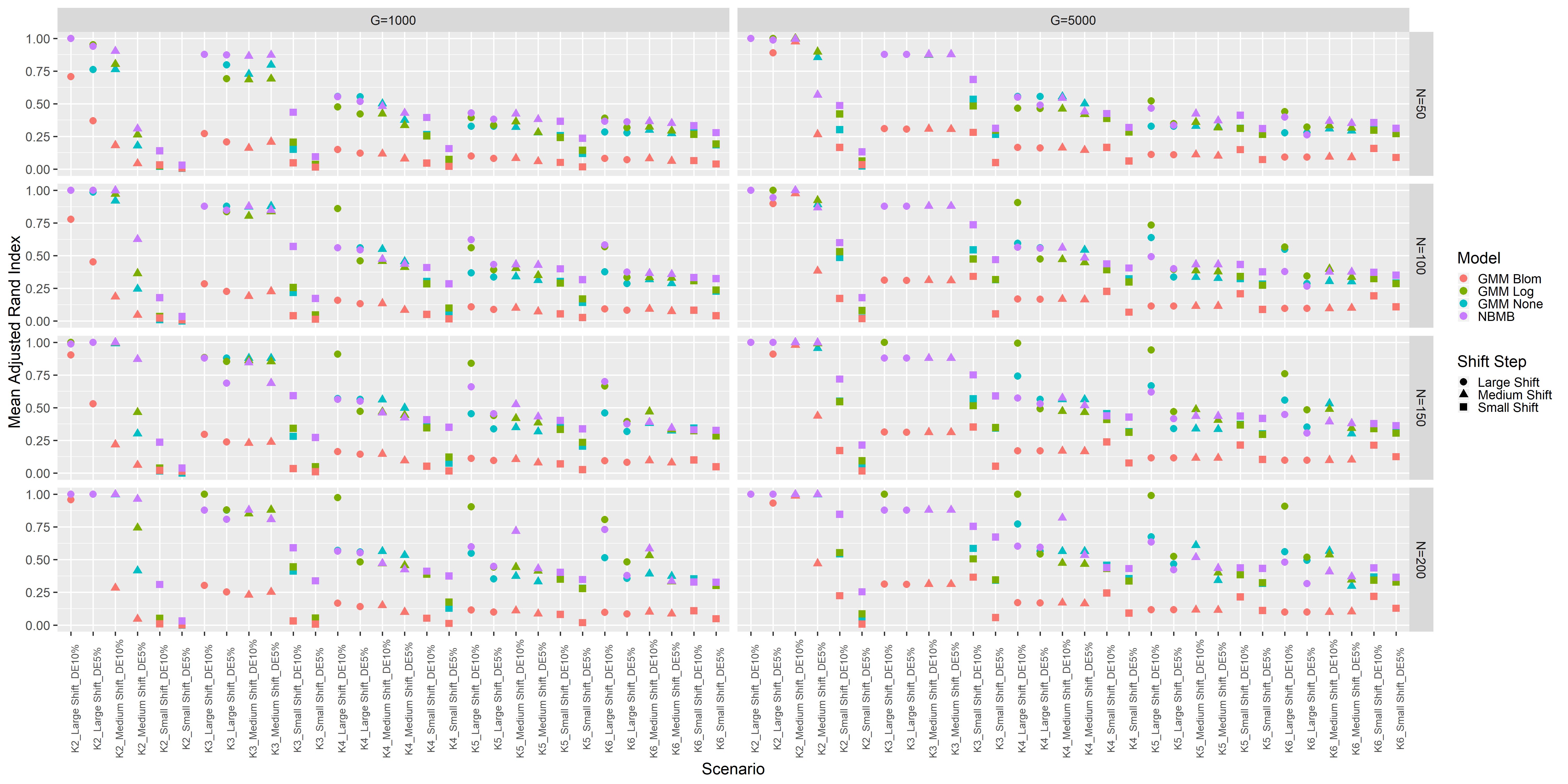

Supplement: Supplementary file 1 — Figure S1. Simulation Results for Zero Shift in Dispersion. Plot of the mean Adjusted Rand Index for 100 simulated datasets in each of the scenarios with zero shift in dispersion parameter. (PNG 246 kb) [file 12859_2018_2556_MOESM1_ESM.png]
